# False negative computed tomography scan due to pelvic binder in a patient with pelvic disruption: a case report and review of the literature

**DOI:** 10.1186/s13256-018-1808-7

**Published:** 2018-09-21

**Authors:** Sharon Jamme, Alexandre Poletti, Axel Gamulin, Olivier Rutschmann, Elisabeth Andereggen, Olivier Grosgurin, Christophe Marti

**Affiliations:** 1 Division of Emergency Medicine, University Hospital of Geneva, 4 rue Gabrielle Perret-Gentil, CH-1211, 14 Geneva, Switzerland; 2 Department of Radiology, University Hospital of Geneva, 4 rue Gabrielle Perret-Gentil, CH-1211, 14 Geneva, Switzerland; 3 Division of Orthopaedic and Trauma Surgery, University Hospital of Geneva, 4 rue Gabrielle Perret-Gentil, CH-1211, 14 Geneva, Switzerland; 4 Division of Internal Medicine, University Hospital of Geneva, 4 rue Gabrielle Perret-Gentil, CH-1211, 14 Geneva, Switzerland

**Keywords:** Pelvic disruption, Trauma, Pelvic binder

## Abstract

**Background:**

Pelvic binders are routinely used in the prehospital setting for stabilization of pelvic injuries in patients with trauma. Emergency department trauma management relies on primary and secondary survey assessment and imaging, most often computed tomography, in hemodynamically stable patients. Maintaining the pelvic binder *in situ* allows stabilization of pelvic injuries during imaging but may hinder the visualization of some pelvic lesions. We report a very rare case of severe pelvic disruption with an absolutely normal computed tomography scan due to the effective placement of a pelvic binder.

**Case presentation:**

We report the case of a 49-year-old Caucasian man referred to our Emergency Department after a high velocity motorcycle accident. Primary assessment revealed a left wrist deformation and pelvic pain, and a pelvic binder was applied by paramedics. A total body computed tomography scan was performed after arrival in our Emergency Department and did not reveal any pelvic injury. The pelvic binder was removed and because of persisting symphyseal pain, pelvic plain radiography was performed revealing a pelvic disruption with an opening of the pubic symphysis and of the left sacroiliac joint (“open book” type pelvic injury) requiring surgical stabilization.

**Conclusions:**

Pelvic binders may mask pelvic disruption in patients with trauma. Pelvic plain radiography should be repeated after pelvic binder removal in patients with high velocity trauma and pelvic symptoms or neurological alterations limiting the reliability of clinical examination.

**Electronic supplementary material:**

The online version of this article (https://doi.org/10.1186/s13256-018-1808-7) contains supplementary material, which is available to authorized users.

## Background

Pelvic fractures occur in approximately 20% of multiple trauma cases, with a mortality rate of up to 20% when associated with hemodynamic instability [1, 2]. Early identification and stabilization of pelvic ring injuries is an important issue in the management of patients with trauma. Historically, the assessment of patients with trauma included manual testing and detection of pelvic instability. However, this maneuver has poor sensitivity and specificity, and might worsen pelvic injuries and is therefore no longer recommended [3].

Current Advanced Trauma Life Support (ATLS) guidelines recommend chest and pelvic X-ray for all patients with blunt trauma. However, different studies suggest that pelvic X-rays have limited value compared with computed tomography (CT) scanning [4, 5] and that pelvic radiography could be eliminated from the primary survey protocol for patients with high-energy blunt trauma who are hemodynamically stable [6, 7]. In these situations, CT with two-dimensional multiplanar reformation (MPR) and three-dimensional reconstructions is the preferred diagnostic strategy.

Pelvic binders have become the preferred method of stabilization for the prehospital management of suspected pelvic ring injuries and are used widely in the management of exsanguinating pelvic trauma. Various reports suggest that they may reduce unstable pelvic fractures and improve hemodynamic status [8–10]. Maintaining the pelvic binder *in situ* may allow stabilization of pelvic injuries during imaging but might also hinder the visualization of some pelvic lesions [11, 12]. Previous cases of false negative CT scans in patients with pelvic disruptions have been reported, but indirect signs such as associated fractures or bleeding were present in these cases. [11, 12] We report a rare case of severe pelvic disruption with an absolutely normal CT scan due to the effective placement of a pelvic binder.

## Case presentation

**Fig. 1:**
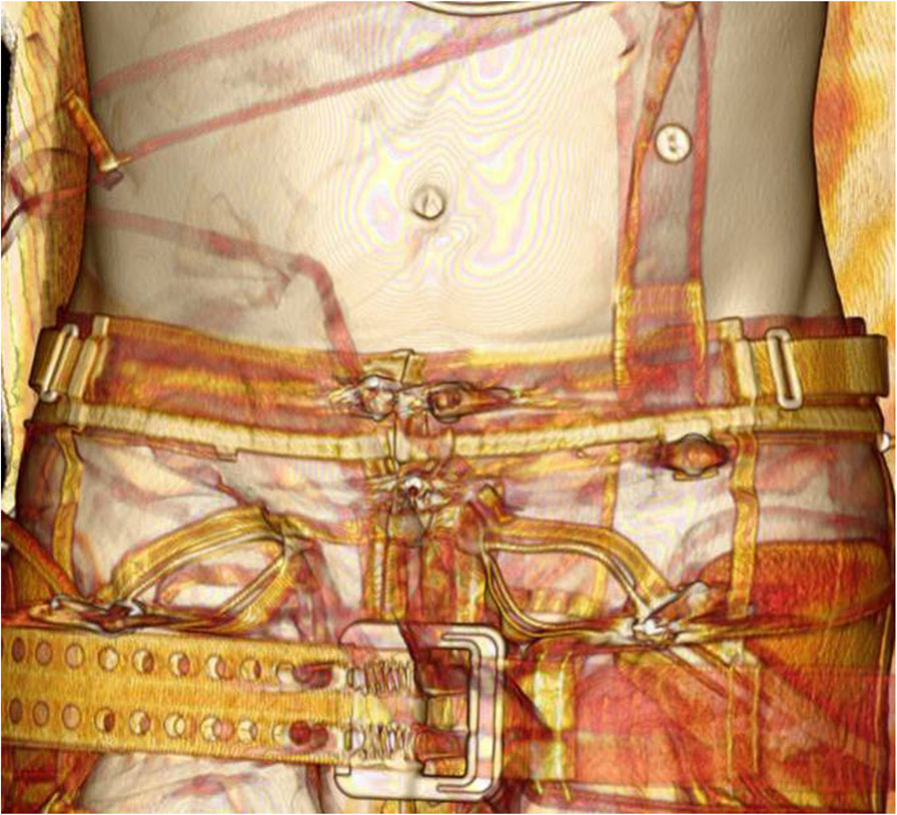
Computed tomography showing the pelvic binder (SAM® Pelvic Sling™ II) *in situ*

A 49-year-old, previously healthy, Caucasian man, a motorcyclist, was referred to our Emergency Department (ED) after a high velocity frontal collision with a car. He was married and worked as a car body repairer. He had no tobacco smoking or drinking history and was not taking any medication. A prehospital primary survey assessment according to ATLS protocols revealed a hemodynamically stable patient with blood pressure (BP) of 136/82 mmHg and heart rate (HR) of 65 beats per minute (bpm) without airway or breathing alterations. He was oriented and conscious and reported pain in the symphyseal region and left arm. Cervical spine immobilization and intravenous access were obtained and a pelvic binder was applied by paramedics (SAM® Pelvic Sling™ II, Fig. 1).

At arrival in our ED, he was alert without any relevant cardiorespiratory dysfunction: body temperature 36.8 °C, HR 65 bpm, BP 132/80 mmHg, oxygen saturation 100%, and Glasgow Coma Scale of 15. A secondary survey revealed a deformation of his left wrist, and painful palpation of the pubic symphysis and sacral region. The pelvic binder was maintained and a total body CT scan with two-dimensional MPR and three-dimensional reconstruction was performed. Laboratory findings revealed mild normocytic anemia (133 g/l) and liver and renal functions were normal. The chronologic timeline of patient management and investigations is provided in the Additional file 1.

**Fig. 2:**
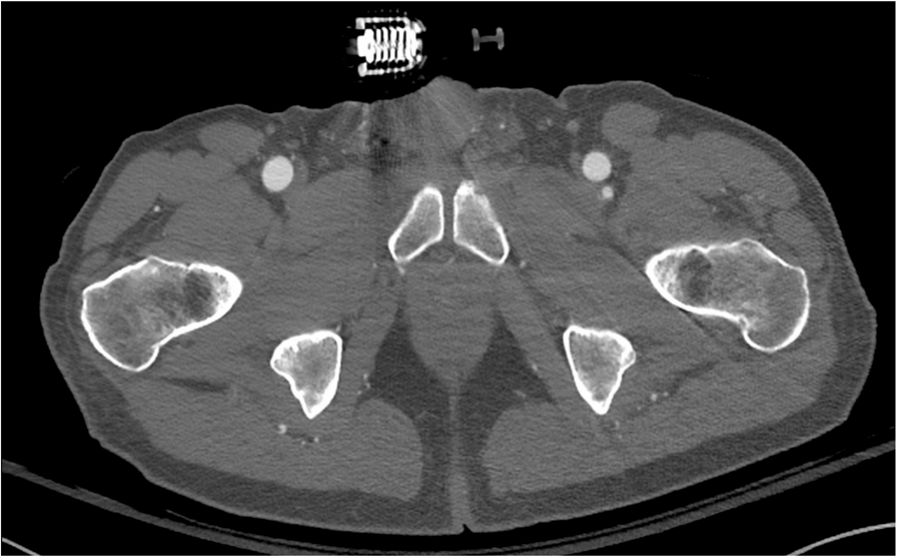
Computed tomography with binder *in situ* showing normal pubic diastasis

**Fig. 3:**
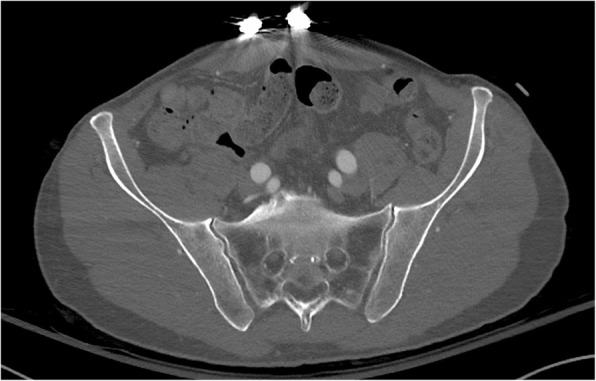
Computed tomography with binder *in situ* showing normal sacroiliac joints

**Fig. 4:**
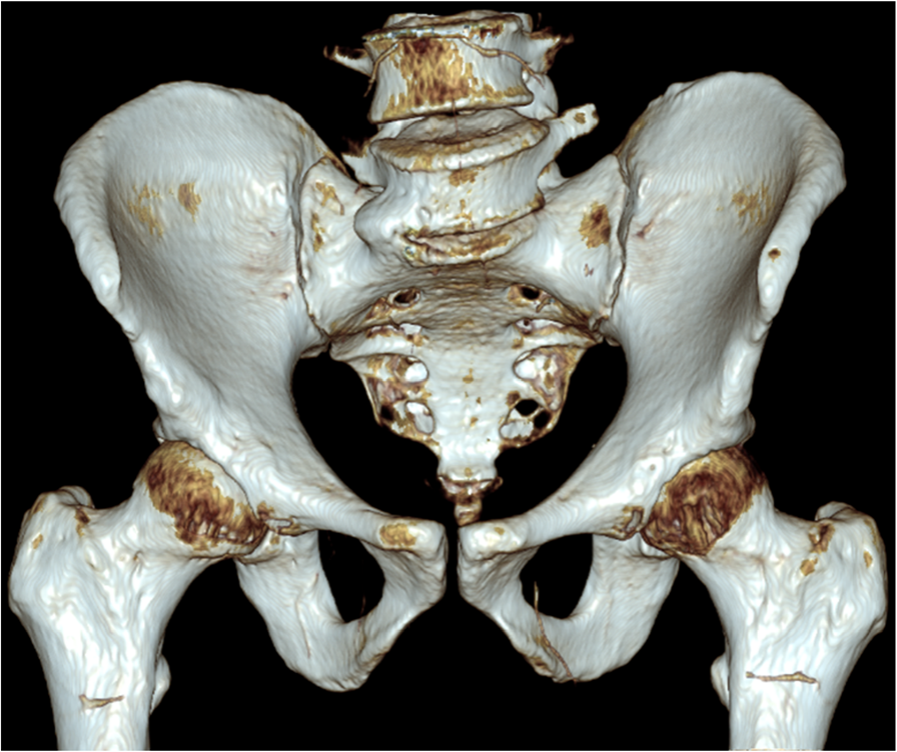
Three-dimensional pelvic reconstruction with pelvic binder *in situ*

No relevant pelvic anomaly was detected (Figs. 2 and 3) including after three-dimensional reconstruction (Fig. 4) and the pelvic binder was removed.

**Fig. 5:**
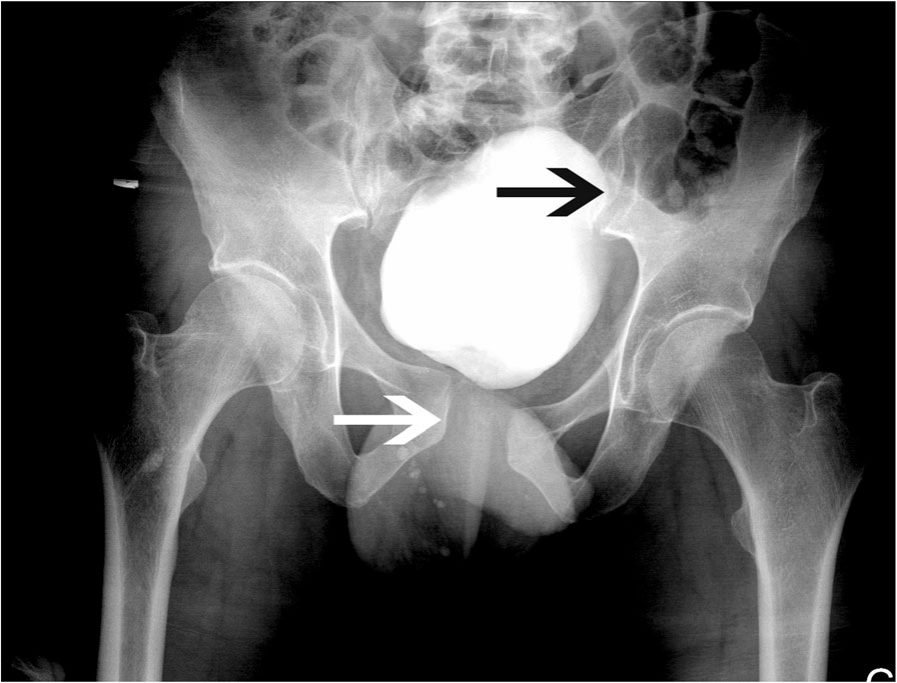
Pelvic anteroposterior plain film after binder removal showing pubic symphysis diastasis (*white arrow*) and left sacroiliac disruption (*black arrow*)

Because of the high velocity of the crash and persisting symphyseal pain, plain anteroposterior pelvic radiography was ordered shortly after the CT. Pelvic radiography revealed a non-osseous pelvic disruption, with an opening of the pubic symphysis (more than 2.2 cm) and of the left sacroiliac joint (type 61-B2.3d according to the 2018 revision of the AO/OTA fracture and dislocation classification compendium; type APC-2 according to the Young and Burgess classification) [13–15] (Fig. 5).

He was admitted to the operating room for surgical pelvic stabilization, and external fixation of his left wrist and was discharged on the tenth day after admission. Full recovery of the pelvic disruption was observed after 6 months and ablation of the left wrist osteosynthesis material was performed at 7 months because of residual pain.

## Discussion and conclusions

In the present case, maintenance of a pelvic binder during radiological imaging resulted in a falsely negative CT scan in a patient with severe pelvic ring disruption. Interestingly, and contrasting with previous report, no indirect sign of pelvic disruption such as associated fracture or bleeding was present, and abdominal and pelvic CT series were interpreted as absolutely normal.

The use of circumferential external compression devices to stabilize potential unstable pelvic fractures was first described in 1999 [16]. Pelvic binders are useful to stabilize pelvic ring injuries and reduce bleeding in the prehospital setting. Cadaveric studies have shown that circumferential sheets and trauma pelvic orthotic devices (PODs) are able to reduce symphyseal diastasis consistently and reduce the symphyseal diastasis to normal in the majority of patients with anterior-posterior compression (APC) type injuries [17]. Moreover, external stabilization using a POD has been shown to be as efficient as external fixation using a pelvic clamp in preventing pelvic displacements in a cadaveric comparative study [18]. Among patients with unstable pelvic fractures, the use of a circumferential external compression device has been shown to reduce transfusion requirements, hospital length of stay, and mortality in a retrospective study [19]. Pelvic binders are quick and easy to apply and are effective in reducing symphyseal diastasis especially in patients with APC-type injuries. However, randomized controlled trials evaluating clinically significant outcomes are lacking.

The effective diastasis reduction obtained by pelvic binder application may, however, hinder the identification of severe pelvic injuries in some patients. Previous cases of effective pelvic reduction using a pelvic binder have been reported [11, 12]. In the majority of these cases, associated lesions such as sacral fractures, extra peritoneal bladder rupture, or lumbar transverse process fractures were identified with CT, whereas, in our case, the abdominal and pelvic CT images were interpreted as absolutely normal.

The present case raises the question of the best diagnostic strategy in patients with suspected pelvic injury. Removal of the pelvic binder before imaging may contribute to worsen hemorrhage in patients with unstable pelvic injuries during CT imaging, while maintaining pelvic stabilization may prevent identifying serious injuries. As imaging with the binder *in situ* will allow identification of the most serious associated lesions for most patients, such as vascular injuries or associated fractures, we suggest that the initial imaging could be performed with the binder in place.

If the initial imaging protocol with the pelvic binder in place fails to identify any sign of pelvic ring injury in patients with high velocity trauma and persistent pelvic symptoms, hemodynamic instability after binder removal, or neurological alterations limiting the reliability of clinical examination, then plain imaging of the pelvis should be systematically repeated after binder removal.

### Conclusion

Pelvic binders may mask pelvic disruption in patients with trauma. Pelvic plain radiography should be repeated after pelvic binder removal in patients with high velocity trauma and pelvic symptoms or neurological alterations limiting the reliability of clinical examination.

## Additional file

Additional file 1: Timeline of patient's evolution and management. (PDF 174 kb)

## Abbreviations

APC: Anterior-posterior compression
ATLS: Advanced Trauma Life Support
BP: Blood pressure
bpm: Beats per minute
CT: Computed tomography
ED: Emergency Department
HR: Heart rate
MPR: Multiplanar reformation
POD: Pelvic orthotic device
