# Supplementary material for: False negative computed tomography scan due to pelvic binder in a patient with pelvic disruption: a case report and review of the literature
Source: J Med Case Rep. 2018 Sep 21;12:271. doi: 10.1186/s13256-018-1808-7 (PMC6149070; doi:10.1186/s13256-018-1808-7)
Supplement: Supplementary file 1 — Timeline of patient's evolution and management. (PDF 174 kb) [file 13256_2018_1808_MOESM1_ESM.pdf]

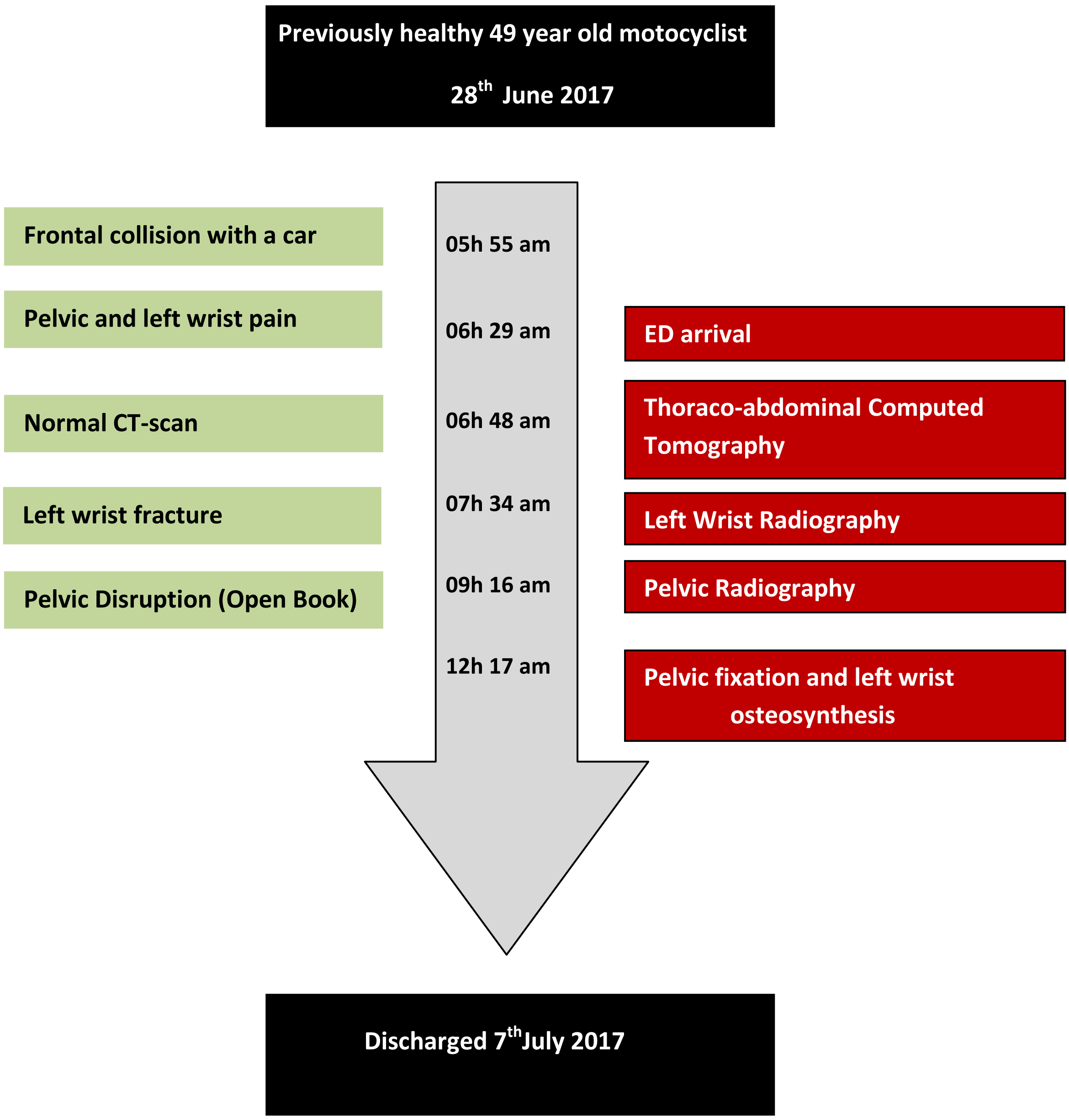

Previously healthy 49 year old motocyclist
28th June 2017
Frontal collision with a car
05h 55 am
Pelvic and left wrist pain
06h 29 am
ED arrival
Normal CT-scan
06h 48 am
Thoraco-abdominal Computed Tomography
Left wrist fracture
07h 34 am
Left Wrist Radiography
Pelvic Disruption (Open Book)
09h 16 am
Pelvic Radiography
12h 17 am
Pelvic fixation and left wrist osteosynthesis
Discharged 7thJuly 2017
